# Assessing Cellular Response to Functionalized α-Helical Peptide Hydrogels

**DOI:** 10.1002/adhm.201400065

**Published:** 2014-03-24

**Authors:** Nazia Mehrban, Edgardo Abelardo, Alexandra Wasmuth, Kieran L Hudson, Leanne M Mullen, Andrew R Thomson, Martin A Birchall, Derek N Woolfson

**Affiliations:** Dr. N. Mehrban, Dr. A. Wasmuth, K. L. Hudson, Dr. L. M. Mullen, Dr. A. R. Thomson, Prof. D. N. Woolfson, School of Chemistry, University of Bristol, Cantock's CloseBristol, BS8 1TS, UK; Dr. E. Abelardo, Prof. M. A. Birchall, University College London Ear Institute, Professorial Unit, Royal Throat, Nose and Ear Hospital330 Grays Inn Rd, London, WC1X 8DA, UK

**Keywords:** 2D cell culture, PC12 cells, peptide hydrogels, self-assembled fibers, tissue engineering

For applications in 2D and 3D cell cultures and tissue engineering, there is a need to develop biocompatible scaffolds that support cell and tissue growth therefore mimicking the biochemical and morphological properties of the natural extracellular matrix (ECM). In order to support cellular growth, the scaffold must provide mechanical stability, promote cellular attachment, proliferation and differentiation, permit diffusion of gases, nutrients and waste and allow control of the degradation rate of the temporary support while minimizing cytotoxic side effects in vivo.^1^

Hydrogels have been extensively investigated and used clinically for cell support in vitro and in vivo in regenerative medicine: their underlying structure mimics the interconnected fibrous network of the ECM;^2^ the hydrated and porous nature of the gels allows diffusion of nutrients into the scaffold and waste to diffuse out^3^,^4^; and bioactive molecules can be incorporated into the fabric of the gels via passive uptake, direct incorporation during material synthesis, or conjugation after synthesis and/or assembly.^5^–^8^

While natural and ex vivo materials such as agarose,^9^ alginate,^10^ carrageenan,^11^ gelatin,^12^ collagen,^13^ and Matrigel^14^ are common current choices for such scaffolds due to their availability and established cellular responses, there is often a lack of control over their formation, degradation, mechanical properties, and chemical modification. Furthermore, ex vivo scaffolds, such as collagens and Matrigel,^15^ show batch-to-batch variation and can potentially introduce disease. Synthetic scaffolds, such as poly(hydroxyethylmethacrylate),^16^ poly(vinyl alcohol),^17^ and polypeptide-based protein anchors^18^ address some of these issues, and provide partially favorable environments for 2D and 3D cell cultures. However, their reduced complexity often fails to mirror native tissue and some degradation by-products can cause unwanted cellular responses.^19^ The advantages and limitations of various tissue engineering scaffolds are reviewed by Chan and Leong.^20^

In principle, bottom-up scaffolds generated and engineered via biomolecular design allow the desired traits from the natural and synthetic scaffolds to be combined into one construct, and so create platforms for guiding cell growth and inducing specific biological responses. With this in mind, a number of peptide-based systems have been reported that utilize amyloid-like assemblies,^21^–^23^α-helical assemblies,^24^–^26^ and peptide amphiphiles^27^–^30^ as building blocks. A challenge in this area is to build complexity and control into these systems, ideally in a modular or pick-and-mix way; some of the systems reported to date lend themselves better to this ambition than others.^31^

Using a bottom-up design approach, we have reported a two-component peptide system for making hydrogels, termed hSAFs (hydrogelating self-assembling fibers).^32^ The peptides (hSAF-p1 and hSAF-p2) are designed de novo using principles for peptide self-assembly. When mixed the two peptides form coiled-coil α-helical fibrous structures, which subsequently interact to form percolated gels. These gels support 2D cell culture. Here, we show that the original two-peptide hSAF system can be supplemented with other components to bring cell-binding functions to the system, hence building up complexity and functionality.

To achieve this functionalization, we developed a variant of hSAF-p1 harboring an azide moiety (**Figure****1**A,B, Figure 1, Supporting Information). This peptide, hSAF-p1(N_3_), was mixed with hSAF-p2 and after overnight gelation an alkyne-bearing peptide containing the cell adhesion motif Arg-Gly-Asp-Ser (alk-RGDS) was added and appended to the hydrogel via copper-catalyzed azide-alkyne cycloaddition (CuAAC; hereafter referred to as the “click reaction”) by overnight reaction in the presence of Cu(I) (Figure B).^33^ Alk-RGDS was used in this study as it promotes cellular attachment via integrin binding.^34^ The use of RGD to promote cellular adhesion in other peptide-based fibrous and hydrogel systems has been reported.^25^,^35^,^36^ We argue here that we gain added utility and control over assembly and functionalization using a modular, dual-peptide system, that is, the α-helical de novo-designed hSAFs. The RGDS-decorated hSAF assemblies were α-helical to an extent comparable to the parent system (see Figure 2, Supporting Information); and electron microscopy (EM) showed that the decoration and subsequent washing procedure did not perturb the gel structure (**Figure** **2**A–D). For this work, we incorporated azidonorleucine at the *N*-terminus of hSAF-p1, although successful decoration was also achieved by substitution at the *C*-terminus (Figure 3, Supporting Information). Gel formation with predecorated p1(N_3_) and p2 was not successful (Figure 4, Supporting Information). Analysis of the decorated gels by high-performance liquid chromatography (HPLC) showed that the RGDS functionality extended entirely through 2 mm thick gels (Figure 5, Supporting Information); and an absorbance-based copper assay showed that the copper used to drive the reaction was successfully removed by subsequent washing (Figure 6, Supporting Information).

**Figure 1:**
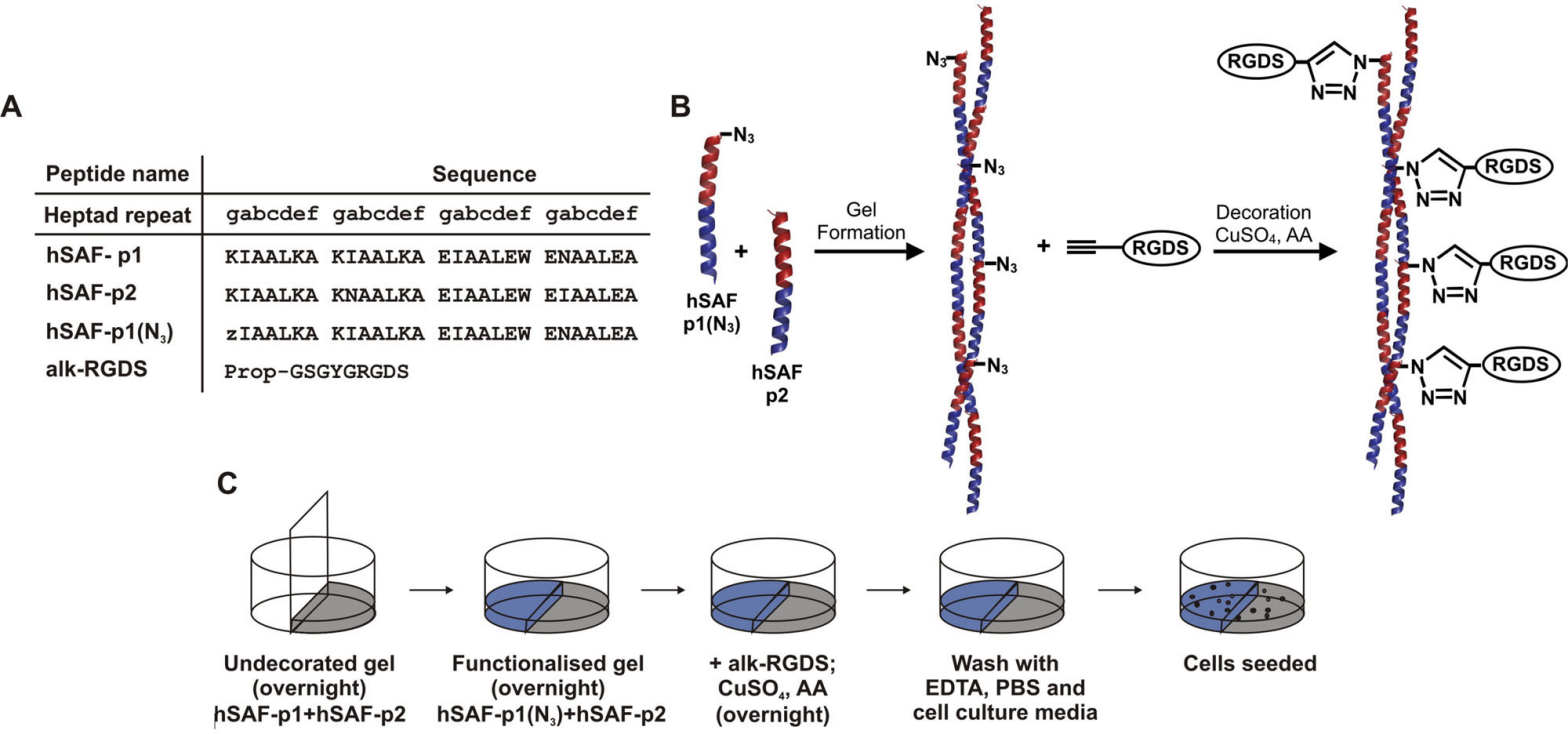
Peptide sequences, a schematic of the click reaction, and the half-moon model. A) Peptide sequences used for this study. Key: z, azido norleucine; Prop, propiolate. B) The gel was formed using an *N*-terminally azido-modified hSAF-p1. Decoration was achieved by performing a click reaction with alk-RGDS on azide-containing gels catalyzed by CuSO_4_ with ascorbic acid (AA). C) Side-by-side gel formation in 24-well cell-culture plates allowed a direct comparison of cellular behavior on undecorated hSAF- and RGDS-decorated hSAF gels. Key: undecorated hSAF gel, gray; and RGDS-decorated hSAF gel, blue.

**Figure 2:**
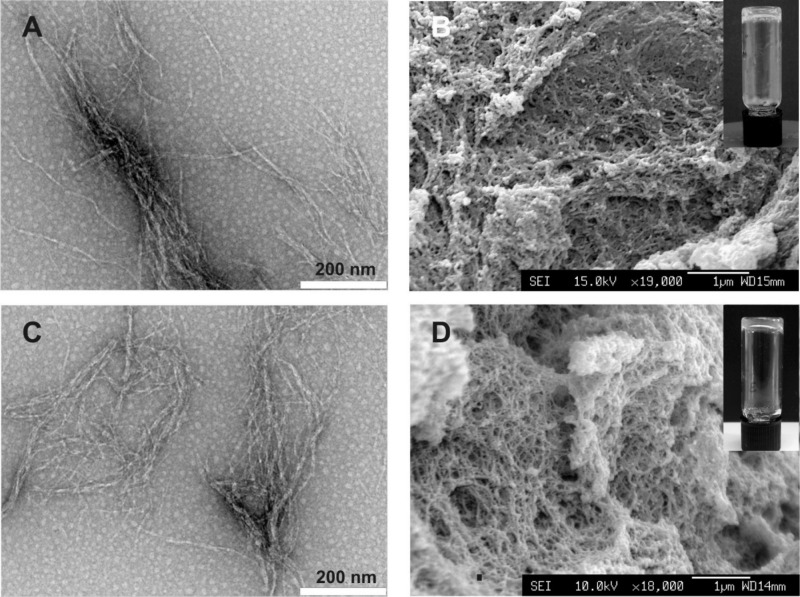
Fiber morphology and gel structure. Transmission electron images for the A) undecorated hSAF and C) RGDS-decorated hSAF fibers. Average fiber diameters were 13 ± 5 nm for hSAF-undecorated fibers and 17 ± 4 nm for RGDS-decorated hSAF fibers. B,D) Scanning electron images showing interconnected fibers forming porous hydrogels of similar morphology D) with and B) without alk-RGDS. The gels are self-supporting (insets). Scale bars on (A,C) equal 200 nm while scale bars on (B,D) equal 1 μm.

To assess and compare cellular responses of undecorated and RGDS-decorated hSAFs, we constructed a “half-moon model” (Figure 1C), in which the two gels were prepared side-by-side in the same tissue-culture well, a similar model to that recently presented by Chan et al.^37^

As a model for neuronal differentiation, we seeded PC12 cells^38^ on both sides of the half-moon hSAF gels. The experiments were followed by light and fluorescence microscopy (**Figure** **3**A–F), and after 14 days cell morphology indicated that cells had attached to both the undecorated hSAF- and RGDS-decorated hSAF sides. However, the number of cells attached to the latter appeared considerably greater (Figure 3A,D).

**Figure 3:**
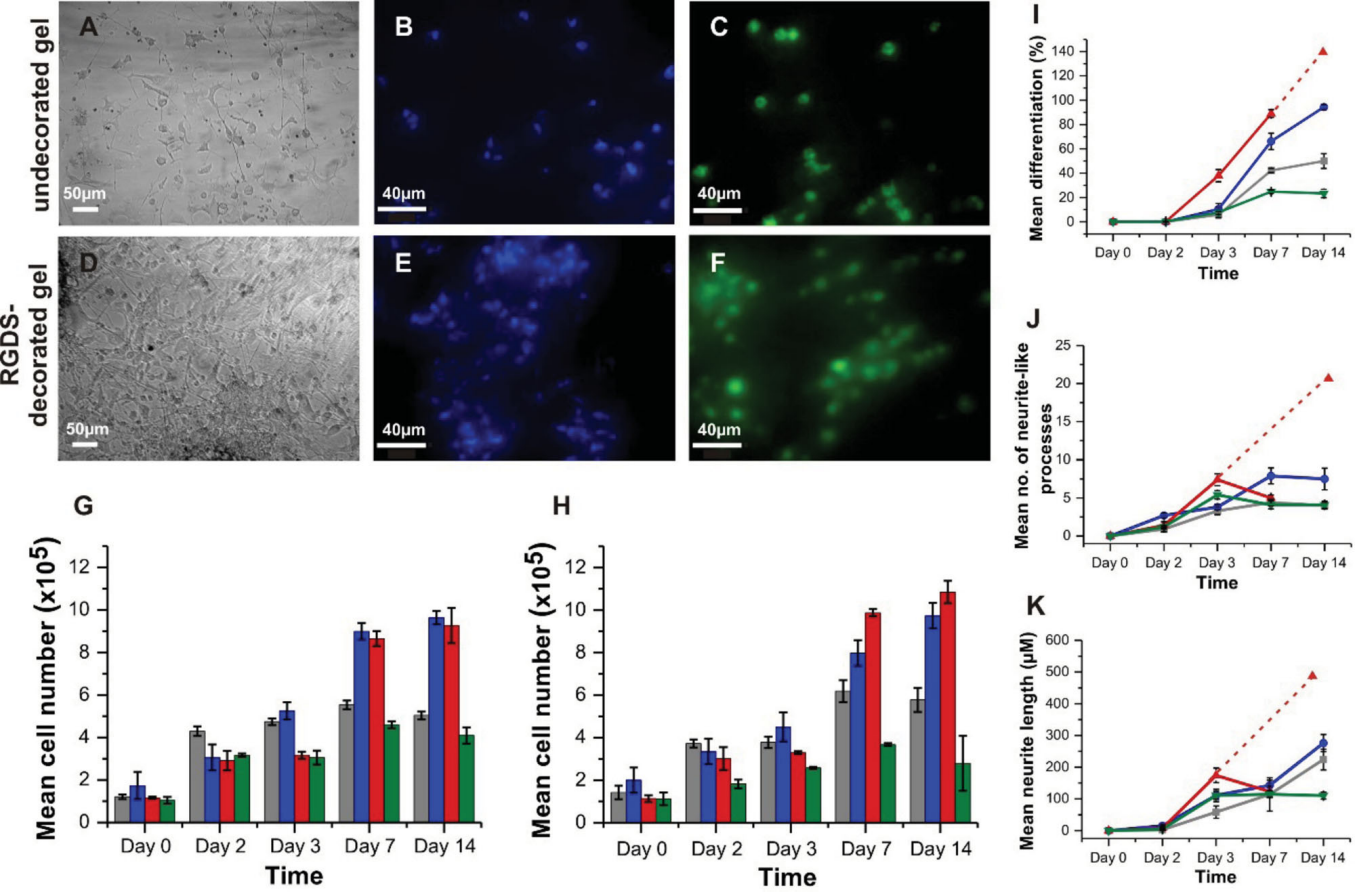
Response of PC12 cells to hydrogels. A,D) Light microscopy images showing PC12 attachment, and elongated cell morphology, to undecorated hSAF- and RGDS-decorated hSAF gels after 14 d. B,E) Representative fluorescent images for DAPI-stained cells on undecorated hSAF- and RGDS-decorated hSAF gels. C,F) Viable cells on undecorated hSAF- and RGDS-decorated hSAF gels indicated by calcein-AM staining. G) Proliferation of PC12 cells on gels and TCP over 14 d as judged by MTT assays. H) DNA quantification using Hoechst dye for PC12 cells on the gels and TCP over 14 d. I) PC12 differentiation, J) number of neurite-like processes, and K) lengths of processes as a function of time. Due to a high proliferation rate, individual cell processes were difficult to identify at day 14 on Matrigel. Dashed lines represent the projections for Matrigel assuming that the underlying trend from the early time points continues. Key: undecorated hSAF gel, gray; RGDS-decorated hSAF gel, blue; Matrigel, red; and TCP, green.

The proliferative activity of the cells growing on the RGDS-decorated side of the gels was ≈50% greater than that of cells on the undecorated side. The higher rates of metabolic activity and proliferation^39^ on the decorated hSAF side were similar to those observed for PC12 cells seeded on commercially available Matrigel (Figure 3G,H; Figures 7 and 8, Supporting Information).

The above-mentioned experiments were conducted without neural growth factor (NGF), which terminates mitosis and induces primary neural outgrowth in PC12 cells.^40^ In parallel experiments, the introduction of NGF promoted cell differentiation, as defined by the presence of neurite-like extensions, by day 3 on both sides of the gel (Figure 3I). Again the degree of differentiation was higher on the hSAF-decorated side: (11 ± 4.6)% cells showed processes on this side, compared with (6 ± 2.6)% on the undecorated side); and the mean number of neural projections extending from the cell body was more than twice as high on the decorated versus undecorated hSAF gel by day 14 (Figure 3J). However, the projection length did not vary significantly between the decorated hSAF and undecorated hSAF halves of the gel (Figure 3K). An assessment of PC12 cells on gels with alk-RGDS attached via the *C*-terminus of hSAF-p1 showed that the effects were similar to those observed with the ligand attached via the *N*-terminus (Figure 9, Supporting Information).

To test the specificity of the peptide–cell interactions, we compared hSAF gels decorated with alk-RGDS and alk-RGES. The latter reduces the efficacy of cell attachment considerably compared with alk-RGDS-based sequences.^41^ We found this to be the case in the hSAF system: changing the aspartic acid (D) to a glutamic acid (E) reduced cellular attachment by approximately 50% (Figure 10, Supporting Information).

The above studies used hSAF gels with every hSAF-p1(N_3_) decorated. It would be advantageous to reduce this percentage to reduce reagent costs and to allow combinations of functionalities to be added via addition of cocktails of modifiers. To begin testing this, we prepared gels with 1%, 10%, and 100% hSAF-p1(N_3_) in hSAF-p1 and performed the click reaction with alk-RGDS. The cellular responses to undecorated hSAF and 1% incorporation were similar. However, the behavior of the 10% and 100% decoration was also similar, showing that considerably less reagent can be used (Figure 11, Supporting Information).

Finally, a separate assessment of 3T3 fibroblast cells with RGDS-functionalized hSAFs showed that although the attachment of the cells appeared greater on RGDS-decorated gels than on the undecorated gels, the proliferative activity of the cells on RGDS-decorated hSAF gels was comparable to that on tissue-culture-treated poly(styrene) (TCP; Figures 12–14, Supporting Information). Thus, not all cells respond significantly to our hSAF gel system.

In summary, we have conjugated a cell-adhesion motif to a rationally designed self-assembling peptide hydrogel system, resulting in stable functional scaffolds suitable for cell culture. Utilization of a “half-moon” protocol allows functionalized and non-functionalized gels to be compared directly in the same tissue-culture well. The morphology, viability, and proliferative activity of PC12 cells seeded on the scaffold surface were demonstrated over 14 days, showing enhanced cellular growth and differentiation on RGDS-modified hSAF gels, highlighting the potential for adding cell-specific motifs to more closely mimic ECM biochemistry. This novel functionalized system offers complex functional scaffolds with tight control over morphology and biochemistry, and with the potential to engineer cell cultures, cell therapy delivery systems, and tissue matrices that closely reflect the in vivo environment and thereby enhance cell performance.

## Experimental Section

*Scaffold Formation*: Peptides were synthesized using standard solid-phase peptide synthesis protocols on a CEM “Liberty” microwave-assisted peptide synthesizer. Peptides were purified by reversed-phase HPLC and their masses confirmed by MALDI-TOF mass spectrometry. Typically, hSAF gels were prepared by mixing separate 1 × 10^−3^ m stock solutions for each parent peptide (hSAF-p1 and hSAF-p2), which were made up in 20 × 10^−3^ m MOPS (3-(*N*-morpholino)propanesulfonic acid) buffer at pH 7.4. This gave final solutions of 0.5 × 10^−3^ m in each peptide. These were left on ice for 5 min followed by 30 min incubation at 20 °C, resulting in gels, which we refer to as 0.5 × 10^−3^ m gels. (n.b., For the *C*-terminally modified peptide, the stock solutions were prepared at 2 × 10^−3^ m, giving “1 × 10^−3^ m gels”.) For decoration experiments, hSAF-p1 was substituted for hSAF-p1(N_3_). After, gel formation was performed by addition of 2 × 10^−3^ m alk-RGDS and CuSO_4_ and ascorbic acid each at 4 × 10^−3^ m final concentration at 20 °C overnight. The gel was then washed with 10 × 10^−3^ m ethylenediaminetetraacetic acid (EDTA) buffer, phosphate buffered saline (PBS), and supplemented-Dulbecco's Modified Eagle Medium (S-DMEM). The presence of remaining copper after decoration was assessed by bicinchoninic acid assay (see Figure 6, Supporting Information). The extent of clicked alk-RGDS was analyzed by analytical HPLC followed, with peak identity confirmed by mass spectrometry. Half-moon gels were formed in 24-well cell-culture plates using sterile glass coverslips as temporary separators for the undecorated hSAF- and RGDS-decorated hSAF gels.

*Biophysical Measurements*: Peptide secondary structure was determined via circular dichroism spectroscopy using a Jasco J-810 CD spectrometer. Fiber morphology was visualized using a JEM 1200 EX MKI transmission electron microscope with a MegaViewII digital camera. Gel scaffold morphology was determined by fixing the sample with glutaraldehyde, removing the moisture via a critical point drying method and imaging using a Jeol JSM-633OF field-emission scanning electron microscope.

*Cell Studies*: PC12 cells, kindly gifted by Prof. Jeremy Henley at the University of Bristol, were seeded onto gels. Cellular morphology was assessed using a light microscope. For live cell imaging, the cells were stained with calcein-AM, their nuclei highlighted with DAPI (4′,6-diamidino-2-phenylindole) and imaged using a Leica DM IRBE inverted epifluorescence microscope. The metabolic activity, and therefore the proliferation rate, of the cells was evaluated by an MTT (3-(4,5-dimethylthiazol-2-yl)-2,5-diphenyltetrazolium bromide) absorbance assay (see §1.14, Supporting Information). These data were supported by a DNA quantification assay (see §1.15, Supporting Information). Differentiated PC12 cells were imaged using light microscopy, and ImageJ was used to count the number of differentiated cells (where differentiation is defined as one or more neural extension being longer than the major diameter of the cell body), the number of extensions per cell and the length of extensions. All quantitative data are presented in the format “mean ± standard error of the mean.” Significant differences between comparable groups were determined by analysis of variance (ANOVA) with post hoc Tukey–Kramer honestly significant difference (HSD). The significance level was set at *p* < 0.05.

## Author Contributions

E.A., M.A.B., N.M., and D.N.W. conceived the project. All authors designed the various experiments. E.A., K.L.H., N.M., A.R.T., and A.W. made the peptides and performed the biophysical work. N.M. and E.A. conducted the cell-culture experiments. M.A.B. and D.N.W. supervised the work. N.M., A.W., and D.N.W. wrote the paper.

## Supporting Information

As a service to our authors and readers, this journal provides supporting information supplied by the authors. Such materials are peer reviewed and may be re-organized for online delivery, but are not copy-edited or typeset. Technical support issues arising from supporting information (other than missing files) should be addressed to the authors.
