# Supplementary material for: Assessing Cellular Response to Functionalized α-Helical Peptide Hydrogels
Source: Adv Healthc Mater. 2014 Mar 24;3(9):1387–91. doi: 10.1002/adhm.201400065 (PMC4276410; doi:10.1002/adhm.201400065)
Supplement: Supplementary file 1 — Supporting Information [file adhm0003-1387-sd1.pdf]

Copyright WILEY-VCH Verlag GmbH & Co. KGaA, 69469 Weinheim, Germany, 2014.

ADVANCED HEALTHCARE MATERIALS

Supporting Information

for *Adv. Healthcare Mater.*, DOI: 10.1002/adhm.201400065

Assessing Cellular Response to Functionalized α-Helical Peptide Hydrogels

Nazia Mehrban, Edgardo Abelardo, Alexandra Wasmuth, Kieran L. Hudson, Leanne M. Mullen, Andrew R. Thomson, Martin A. Birchall, and Derek N. Woolfson**

Supporting Information

for *Adv. Mater.*, DOI: 10.1002/**adhm.201400065**

Title Assessing Cellular Response to Functionalized α-Helical Peptide Hydrogels.

Nazia Mehrban, Edgardo Abelardo, Alexandra Wasmuth, Kieran L. Hudson, Leanne M. Mullen, Andrew R. Thomson, Martin A. Birchall, Derek N. Woolfson**

Supporting Information

1.0 Methodology

1.1. Materials

Unless otherwise specified, standard protocols were used. Reagents were purchased from Sigma Aldrich (Gillingham, UK), Rathburn Chemicals (Walkerburn, UK) or Fisher Scientific (Loughborough, UK). Fmoc-protected amino acids, *O*-(7-azabenzotriazol-1-yl)-*N*,*N*,*N'*,*N'*-tetramethyluronium hexafluorophosphate (HBTU) and peptide-grade dimethylformamide (DMF) were purchased from AGTC Bioproducts (Hessle, UK.).

1.2 Peptide synthesis

Peptides were synthesized on H-Ala-HMPB-ChemMatrix resin (PCAS BioMatrix Inc., Saint-Jean-sur-Richelieu, Canada) on a 0.5 mmol scale with a `Liberty' microwave assisted peptide synthesizer (CEM, Buckingham, UK) using standard 9-fluorenyl-methoxycarbonyl (FMOC)-based solid-phase chemistry with HBTU activation. α-fmoc-ε-azido-norleucine was manually coupled using 2 eq. amino acid, 1.9 eq. *O*-(7-azabenzotriazol-1-yl)-*N*,*N*,*N'*,*N'*-tetramethyluronium hexafluorophosphate (HBTU) and 2.5 eq. *N*,*N*-Diisopropylethylamine. For the alkyne modification of RGDS 5 eq. propiolic acid, 6 eq. hydroxybenzotriazole and 4.5 eq. *N*,*N'*-diisopropylcarbodiimide were used. Cleavage of the peptides with complete removal of the side-chain protecting groups was achieved by incubation at 20 °C with trifluoroacetic acid (TFA)/triisopropylsilane/water (38:1:1, 3 h). Cleaved products were precipitated in cold diethyl ether, centrifuged, and the precipitate was dissolved in 5 mL of a 1:1 mixture of acetonitrile/water and then lyophilized to give peptides as fine white solids.

1.3 Peptide purification

All peptides were purified by reverse-phase high-performance liquid chromatography (HPLC; JASCO, Great Dunmow, UK) using a Vydac® TP C18 column (10 µm, 22 × 250 mm) under acidic conditions (buffer A: 0.1% TFA in H_2O; buffer B: 0.1% TFA in acetonitrile). Depending on the peptide sequence, different gradients were used. The peptides hSAF-p1, hSAF-p1(N_3) and hSAF-p2 were purified using a 20 to 60% buffer B gradient over 60 min, alk-RGDS was purified using a 5 to 40% buffer B gradient over 60 min. Fractions containing pure peptides were lyophilized subsequently.

1.4 Peptide characterization

Peptide masses were confirmed by matrix-assisted laser desorption/ionization-time of flight mass spectrometry (MALDI-TOF MS) on a Applied Biosystems 4700 Proteomics Analyzer MALDI-TOF instrument (matrix: α-cyano-4-hydroxycinnamic acid (CHCA), external calibration). All peptides were of >95% purity as monitored by analytical HPLC (JASCO, Great Dunmow, UK) using a Phenomenex Prodigy ODS-3 column (5 µm, 4.6 × 100 mm). Peptide concentrations were determined by UV absorbance (ε_{280}(Trp) = 5690 mol^{-1} cm^{-1}, ε_{280}(Tyr) = 1280 mol^{-1} cm^{-1}) using a NanoDrop 2000 spectrophotometer from Thermo Scientific.

1.5 Scaffold formation

hSAF gels were prepared by dissolving the complementary peptides (hSAF-p1, hSAF-p1(N_3) and hSAF-p2) in 3-(N-morpholino)propanesulfonic acid (MOPS)-buffer (20 mM MOPS, 5 mM sodium acetate, 1 mM EDTA) pH 7.4, at 2 mM (approximately 3 mg mL^{-1}) of each peptide. The peptide solutions were mixed and set on ice for 10 min, followed by overnight at 20 °C. Undecorated hSAF gels consisted of peptide hSAF-p1 and hSAF-p2 whilst decorated hSAF gels contained hSAF-p1(N_3) and hSAF-p2. To decorate the gel was performed by pre-mixing 4 mM $CuSO_4$, 4 mM ascorbic acid and 2 mM of alk-RGDS and incubating of the

azide containing gel with this mixture overnight at 20 °C. To remove residual copper salts, the gel was washed 10 times with 10 mM EDTA, 10 times with phosphate buffered saline (PBS; 160 mM NaCl, 3 mM KCl, 8 mM $NaHPO_4$, 1 mM KH_2PO_4, pH 7.4) and perfused with supplemented media (S-DMEM) before the cell seeding experiments.

Half-moon gels were formed in 24-well tissue-culture plates (CellStar, Greiner Bio-one Ltd, UK) using sterile glass coverslips as temporary separators mounted vertically in the middle of the wells. One half of the well was filled with the undecorated hSAF gel solution and incubated overnight at 37 °C. The next day the coverslip was removed and the second half of the well filled with the decorated hSAF peptide mix. The gel was again incubated overnight at 37 °C, 5% CO_2 and 100% relative humidity.

1.6 Degree of decoration

To confirm that the reaction was successful, a sample of the hSAF decorated gel was mixed in 20:80 v/v water-acetonitrile buffer containing 0.1% v/v trifluoroacetic acid (TFA) to disassemble the gel. HPLC was used in analytical mode with a 30 to 40% buffer B gradient over 50 min to conduct an area under the curve (AUC) analysis, thus determining the ratio of functionalized to non-functionalized peptide. Peaks were collected and masses were confirmed by MALDI-TOF mass spectrometry. All clicked samples were compared to controls containing no $CuSO_4$, ascorbic acid or alk-RGDS.

1.7 Copper Assay

Copper levels in washed hSAF decorated gels was assessed by modification of a previously published colorimetric procedure, where the presence of copper is detected by the formation of a complex with distinctive absorbances at 356 and 562 nm.[1] 50 µL Trichloroacetic acid (300 g L^{-1} in H_2O) was added to 50 µL of the gel and the suspension was transferred to a 0.5

mL microfuge tube and vortexed. The precipitate was removed by centrifugation (10,000 rpm, 10 minutes) and 50 µL of the supernatant transferred to a fresh 0.5 mL microfuge tube. To this 10 µL of L-gulonic acid γ-lactone (352 mg L^{-1} in H_2O) was added and the solution vortexed. 40 µL of a solution containing bicinchoninic acid disodium salt (67 mg L^{-1}), NaOH (40 g L^{-1}) and N-(2-hydroxyethyl)piperazine-N′-(2-ethanesulfonic acid) (HEPES buffer) anhydrous (173 g L^{-1}) was added and the mixture vortexed again. The solution was left for 2 hours at 20 °C, and the absorbance measured at 355 nm on a Perkin Elmer Lambda 25 UV/Vis spectrometer with a path length of 1 cm and the equivalent measurement for a blank containing reagents but no peptides subtracted.

1.8 Circular dichroism (CD) spectroscopy

Protein secondary structures were assessed *via* CD spectroscopy using a using a JASCO J-810 spectropolarimeter fitted with a Peltier temperature controller. CD spectra were recorded at 100 µM total peptide concentration in MOPS in 0.1 cm quartz cuvettes from Starna. Measurements were performed at 20 °C, 190 and 260 nm with a 1 nm data pitch and bandwidth, 4 s response time, 50 nm min^{-1} scanning speed and averaged over 2 accumulations. Baselines were recorded using the same buffer, cell and parameters as the samples and subtracted from the data.

1.9 Transmission electron microscopy of fibers

3 µL samples were spotted on a carbon-coated 400-mesh copper grids (Agar) on filter paper, air-dried overnight at 20 °C and stained with 3 µL 1% w/v uranyl acetate in H_2O. Images of the fibrils were viewed on a JEM 1200 EX MKI transmission electron microscope (JEOL, UK) at an accelerating voltage of 120 kV and captured using a MegaViewII digital camera (Olympus Soft Imaging Systems GmbH, Germany).

1.10 Scanning electron microscopy

Scaffold morphologies of the hSAF gels were assessed by first fixing the samples using 2.5% v/v glutaraldehyde (in 0.1 M PBS in H_2O) for 1 h at 4 °C and dehydrating by exchange with graded ethanol 20%, 30%, 50%, 70%, 90% and 100% v/v ethanol in H_2O for 10 min each. The ethanol in the samples was then exchanged with liquid CO_2 in (Galaxy S+, RS Biotech, Eppendorf UK Limited, UK), which was then slowly evaporated at 1070 psi and 31 °C. Finally, the specimens were coated with gold by a sputtering method using 25 mA and 1.5 kV (EmiTech K575X Sputter Coater, Quorum Technologies Ltd, UK); the thickness of sputtered gold was between 10 and 12 nm. A Jeol JSM-633OF field emission scanning electron microscope (FEG-SEM, UK) was used at an operating voltage of 20 kV, a working distance of 15 mm and spot size 3 to obtain images.

1.11 Cell culture

Cells derived from the pheochromocytoma of the rat adrenal medulla (Cellomics, Thermo Fischer, Belgium) were cultured in supplemented DMEM with 10% v/v horse serum, 5% v/v FBS, 1% v/v pen-strep and 2.5% v/v L-glutamine. After 24 h the media was replaced with fresh supplemented media containing 1% v/v horse serum and 0.5% v/v FBS. Differentiation was induced by adding 100 ng mL^{-1} nerve growth factor (NGF, Sigma-Aldrich, UK).

Murine fibroblasts (NIH 3T3, ATCC, UK) were cultured in supplemented high glucose DMEM (Sigma-Aldrich, UK) with 10% v/v fetal bovine serum (FBS, PAA, UK), 1% v/v penicillin-streptomycin (pen-strep, Sigma-Aldrich, UK), 2.5% v/v L-glutamine (Sigma-Aldrich, UK) and 2.5% v/v 1 M 4-(2-hydroxyethyl)-1 piperazineethane-sulfonic acid (HEPES buffer, Sigma-Aldrich, UK).

All cells were maintained at 37 °C, 5% CO_2 and 100% relative humidity with the media being changed every 3 days. Cells were passaged using trypsin (Sigma-Aldrich, UK), with cells at passage 5 being used for this study.

1.12 Surface seeding

Trypsinized cells were counted using trypan blue (Sigma-Aldrich, UK) stained cells on a haemocytometer. Gels were made as previously outlined in 96-well or 24-well tissue-culture plates. The gelled discs were seeded with 1.0 x 10^4 cells and supplemented with 100 μL supplemented media. The seeded samples were incubated at 37 °C, 5% CO_2 and 100% relative humidity (Galaxy S+, RS Biotech, Eppendorf UK Limited, UK) and the media changed every 3 days.

1.13 Light and fluorescence microscopy

Cell samples were washed with PBS on days 0, 3, 7 and 14 post-seeding. For live-cell imaging, 7 μL calcein-AM was added to the sample and imaged using a Leica DM IRBE inverted epifluorescence microscope (Leica, UK). To obtain DAPI (Sigma-Aldrich, UK) stained nuclei, cells were first fixed for 2 h with 4% w/v glutaraldehyde in H_2O (Sigma-Aldrich, UK) post-washing and stained with 50 μL DAPI dye. The sample was incubated at 37 °C for 5 min and washed 3 times with PBS, immersed in fresh PBS and imaged using the aforementioned microscope. 6 areas (228 μm x 174 μm) on DAPI stained samples were imaged, the average values of which have been presented.

1.14 Metabolic activity assay using MTT (3-(4,5-dimethylthiazol-2-yl)-2,5-diphenyltetrazolium bromide)

The metabolic activity, and therefore proliferation rate, of surface-seeded cells on days 0, 3, 7 and 14 post-seeding was evaluated by adding 3 μL MTT (10% tetrazolium MTT, Sigma Aldrich, UK, in PBS) to a cellular 30 μL gelled scaffold with supplemented media not

containing NGF. After 18 h incubation at 37 °C, 200 μL HCl-isopropanol (1:24) was added to the well and incubated for a further 45 min at 37 °C. The absorbance of the resulting solution was measured in triplicate, at 620 nm (SpectraMax M2, Molecular Devices, USA) and used to calculate the number of metabolizing cells present in each sample.

1.15 DNA quantification

Double-stranded DNA in gel/cell samples on days 0, 3, 7 and 14 post-seeding was quantified by freezing the cellular samples not containing NGF at -80 °C and adding 100 μL sterile de-ionized water before incubating at 37 °C for 1 h. A solution was prepared by adding 25 μL Hoechst stain (Hoechst 33258, Sigma-Aldrich, UK) to 10 mL TNE buffer (100 mM Tris base, 2 M NaCl, 10 mM EDTA) with 90 mL 18.2 MΩ.cm ultrapure water and adjusted to pH 7.4. 100 μL of the solution was added to each of the thawed samples. Samples were excited at 365 nm and the fluorescence measured at 450 nm. A calibration curve based on the fluorescence of cells with increasing seeding density was prepared to approximate the cell number for the study.

1.16 Quantification of PC12 differentiation, neurite-like extensions and length of cytoplasmic projections

Differentiated PC12 cells, their neurite-like extension number and extension length were identified and counted using ImageJ software (ImageJ 1.46r, USA) for half-moon samples. Measurements were taken at day 0 (after 3 h), 1, 3, 7, 14 from 3 microscopic images taken from different locations over a single sample on each day.

1.17 Statistical analysis

Data are presented in the format "mean ± standard error of the mean". Differences in mean values were compared within groups and significant differences were determined by ANOVA

(Analysis Of Variance) with post-hoc Tukey-Kramer HSD (honestly significant difference) test. The significance level was set at $p < 0.05$.

2.0 References

[1] A. J. Brenner and E. D. Harris, *Anal. Biochem*. **1995**, *226*, 80-84.

3.0 Additional Figures

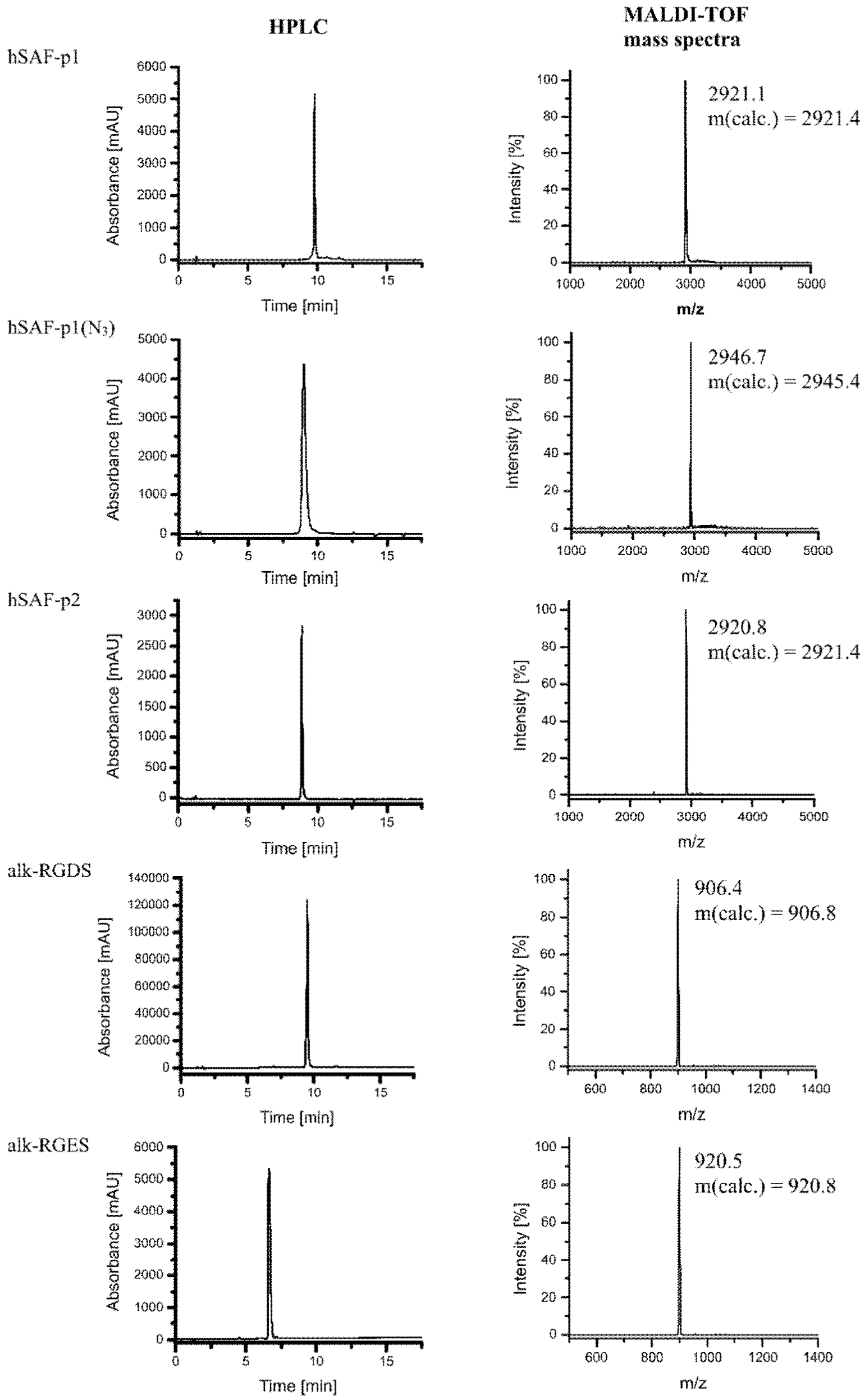

Figure S1. High performance liquid chromatograms and mass spectra for all peptides used in this study. Peptide purity was assessed by analytical HPLC and the peptide mass confirmed by MALDI-TOF mass spectrometry. Key: m(calc.), calculated masses of the peptides.

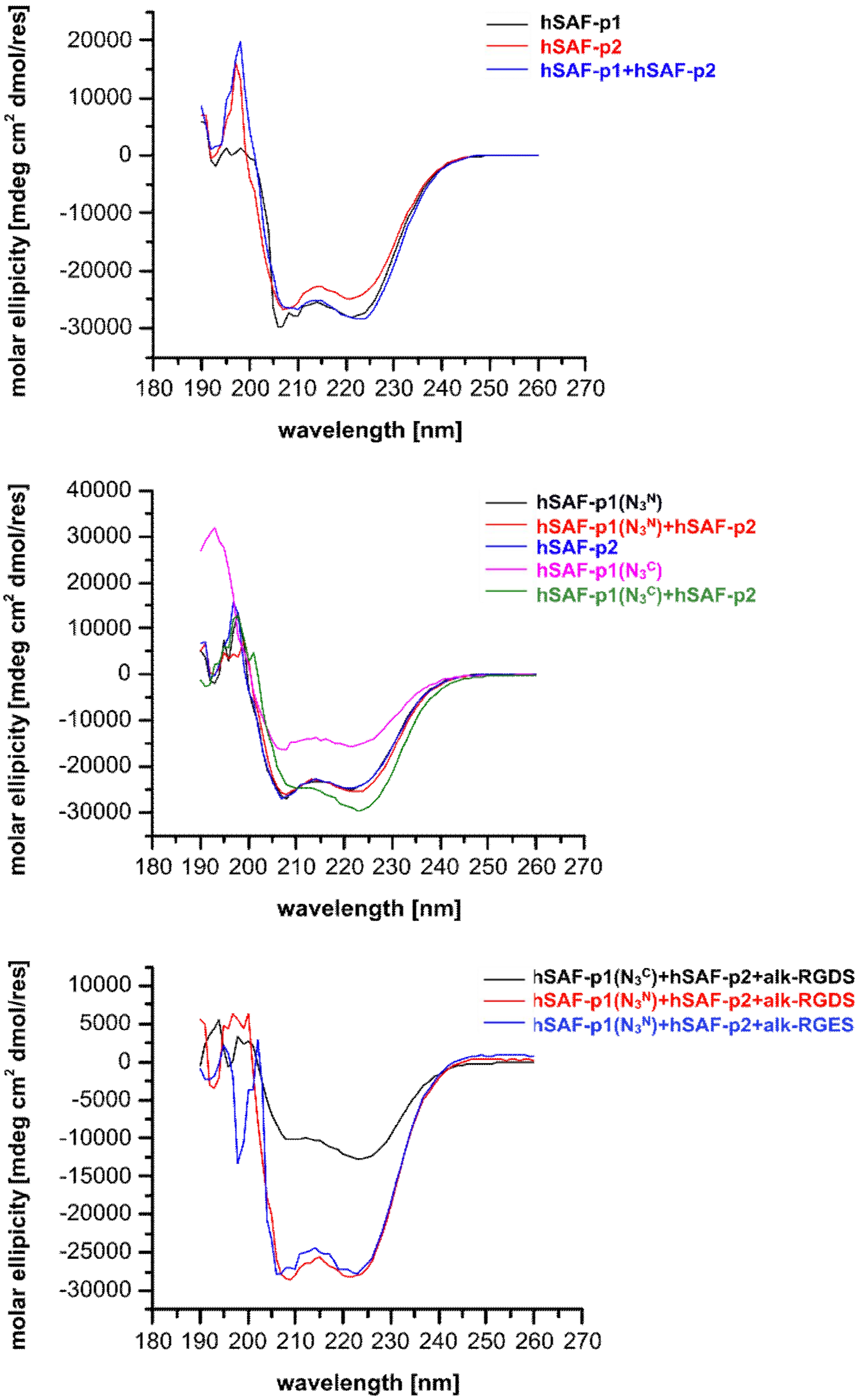

Figure S2. Peptide secondary structure. CD spectra of all peptides used for this study. These show that all peptides are predominantly α-helical. Key: ^{N}, *N*-terminus modified peptides; ^{C}, *C*-terminus modified peptides.

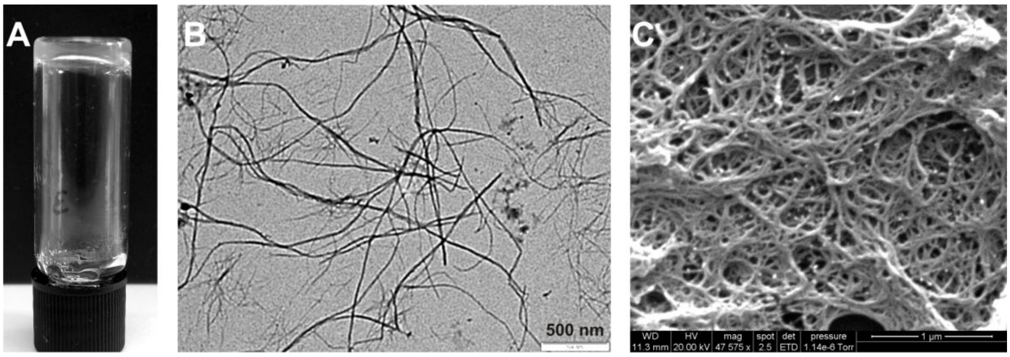

Figure S3. Gel formation and electron micrographs showing scaffold morphology of *C*-terminally decorated hSAF fibers (1 mM each peptide). The *C*-terminally decorated gels (A) were composed of fibers (B) as observed by TEM with diameter 5-10 nm resulting in a porous scaffold (C) observed by SEM.

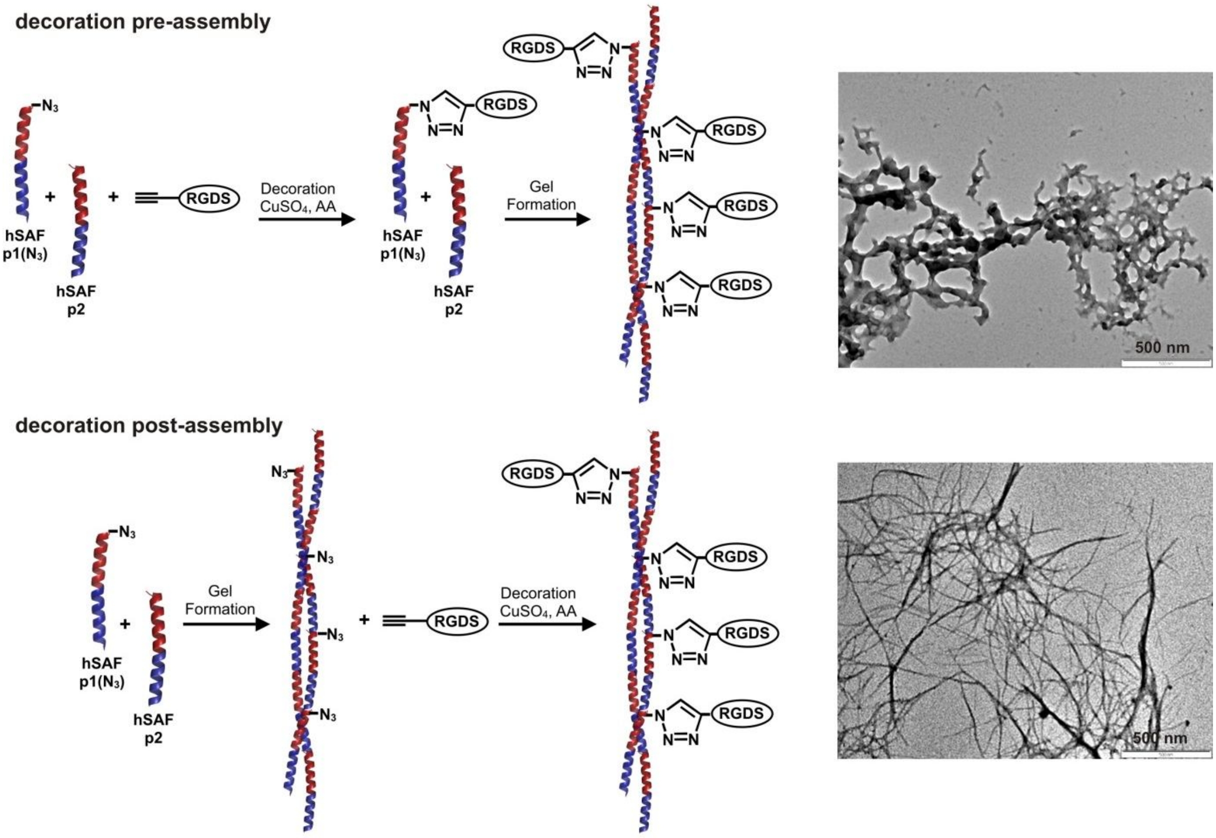

Figure S4. Methods of decorating the hSAF fibers and gels. Schematics and transmission electron micrographs showing the morphology of hSAF fibers decorated pre-assembly and post-assembly with alk-RGDS. The latter results in better fiber morphology and, consequently, gel formation.

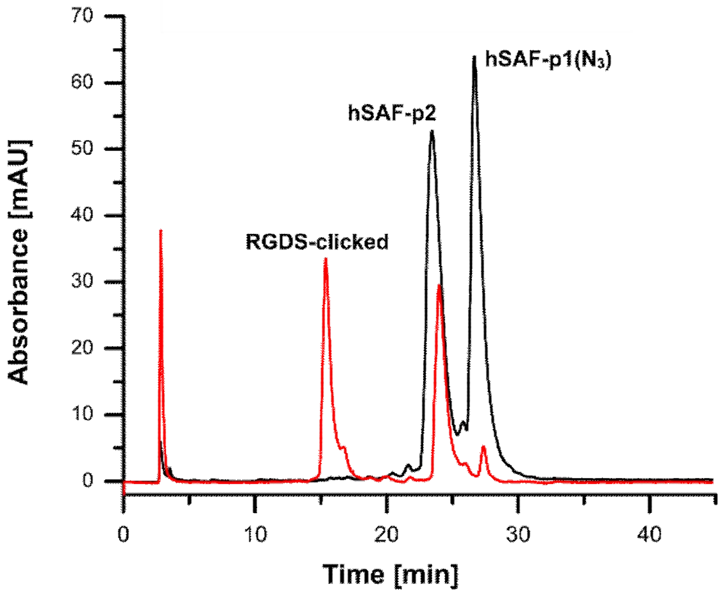

Figure S5. Analysis of the click reaction. HPLC comparison of undecorated hSAF (black) and RGDS-decorated hSAF (red) gels showed that the majority (90%) of the hSAF-p1(N_3) is clicked in an overnight reaction. The minimum percentage of decorated hSAFs is 39%, based on the expected number of modifiable coiled coils on the surface of a fiber with a 14 nm diameter as determined by TEM.

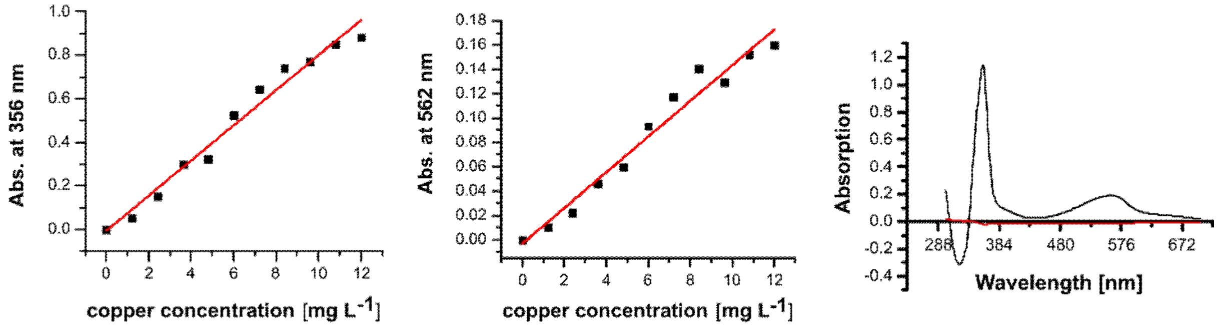

Figure S6. Analysis of copper levels present in gel after the click reaction. Calibration curves for the copper assay at 356 nm and 562 nm with the absorption spectra of gel samples showing the absorbance of gel treated with copper, but unwashed (black) and gel treated with copper and washed (red).

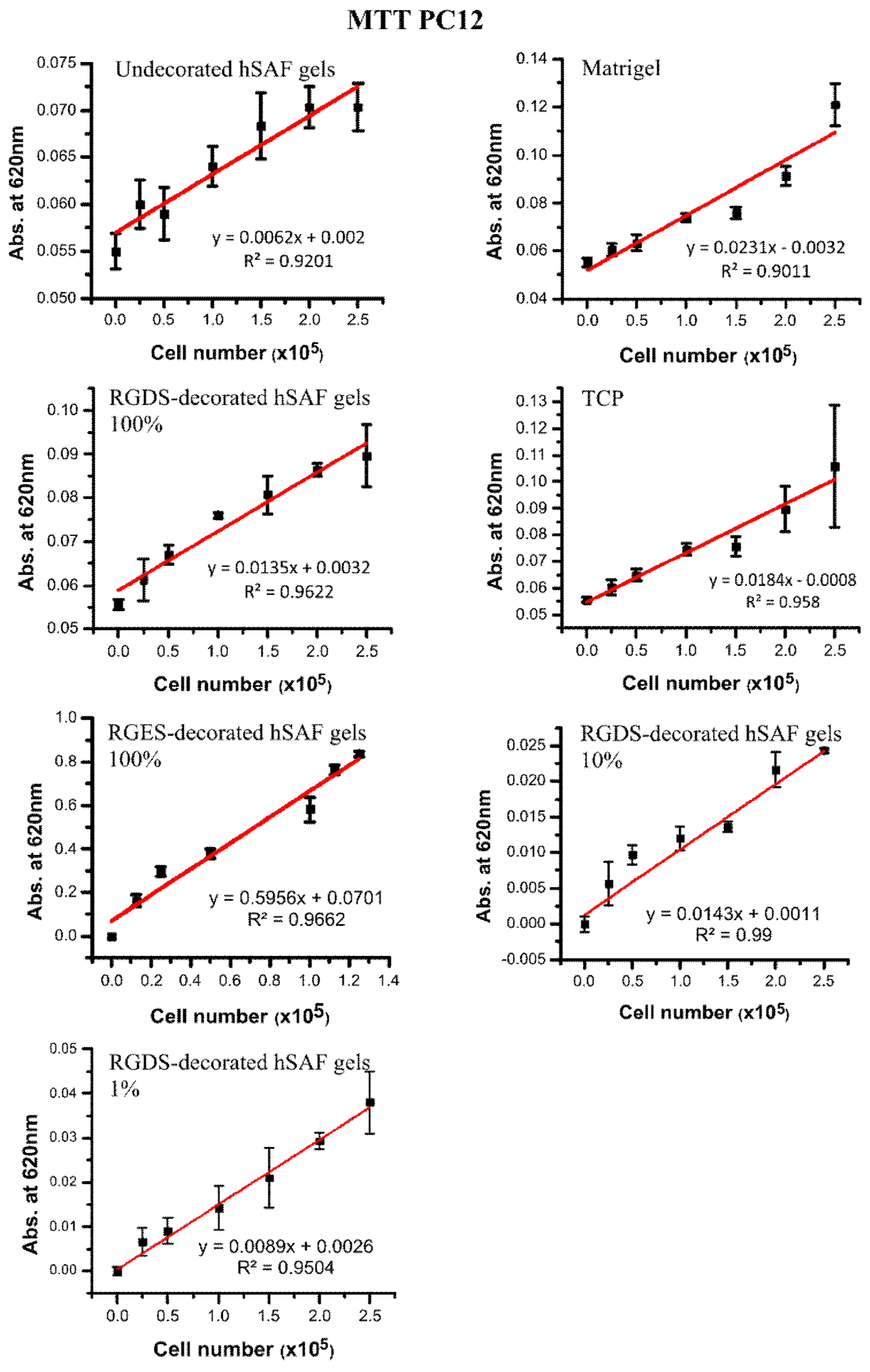

Figure S7. Metabolic activity for PC12 cells. MTT was used to assess the metabolic activity of PC12 cells on undecorated hSAF gels, Matrigel, 100% RGDS-decorated hSAF gels, tissue-culture plastic, 100% RGES-decorated hSAF gels, 10% RGDS-decorated hSAF gels, and 1% RGDS-decorated hSAF gels. Proliferative activity of the cells was then calculated from the

calibration curves.

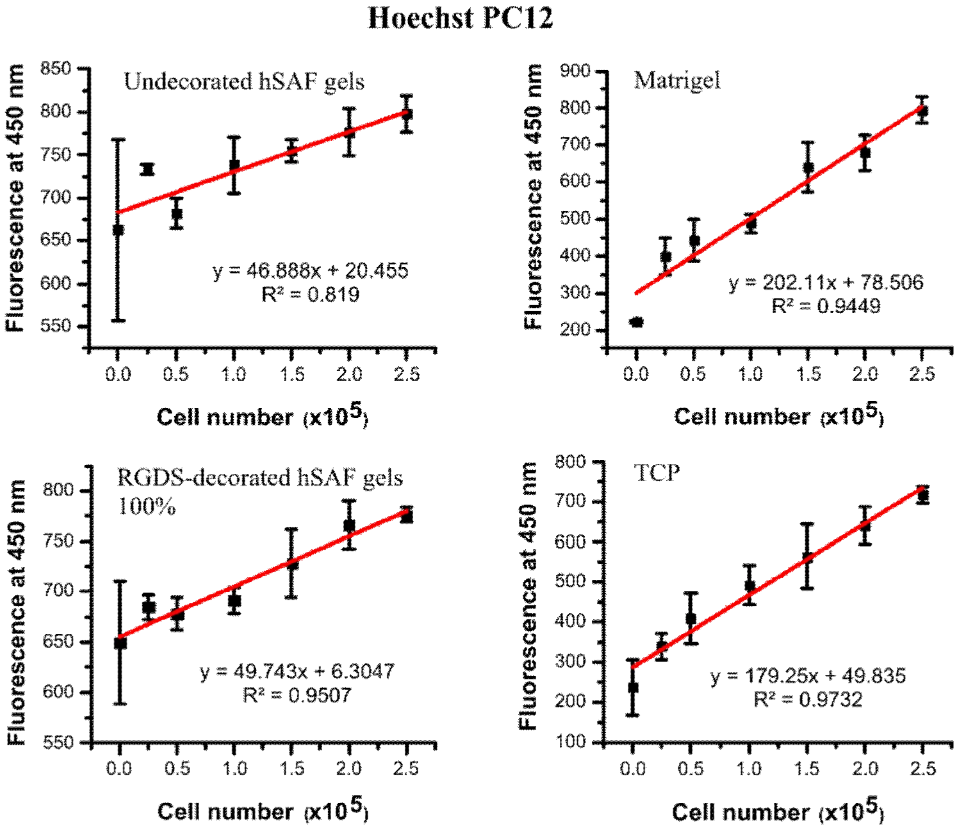

Figure S8. DNA quantification of PC12 cells using Hoechst dye. Calibration curves of PC12 cells stained with Hoechst dye were used to quantify the DNA content and assess approximate proliferative activity of the cells on undecorated hSAF gels, Matrigel, 100% RGDS-decorated hSAF gels, and tissue-culture plastic.

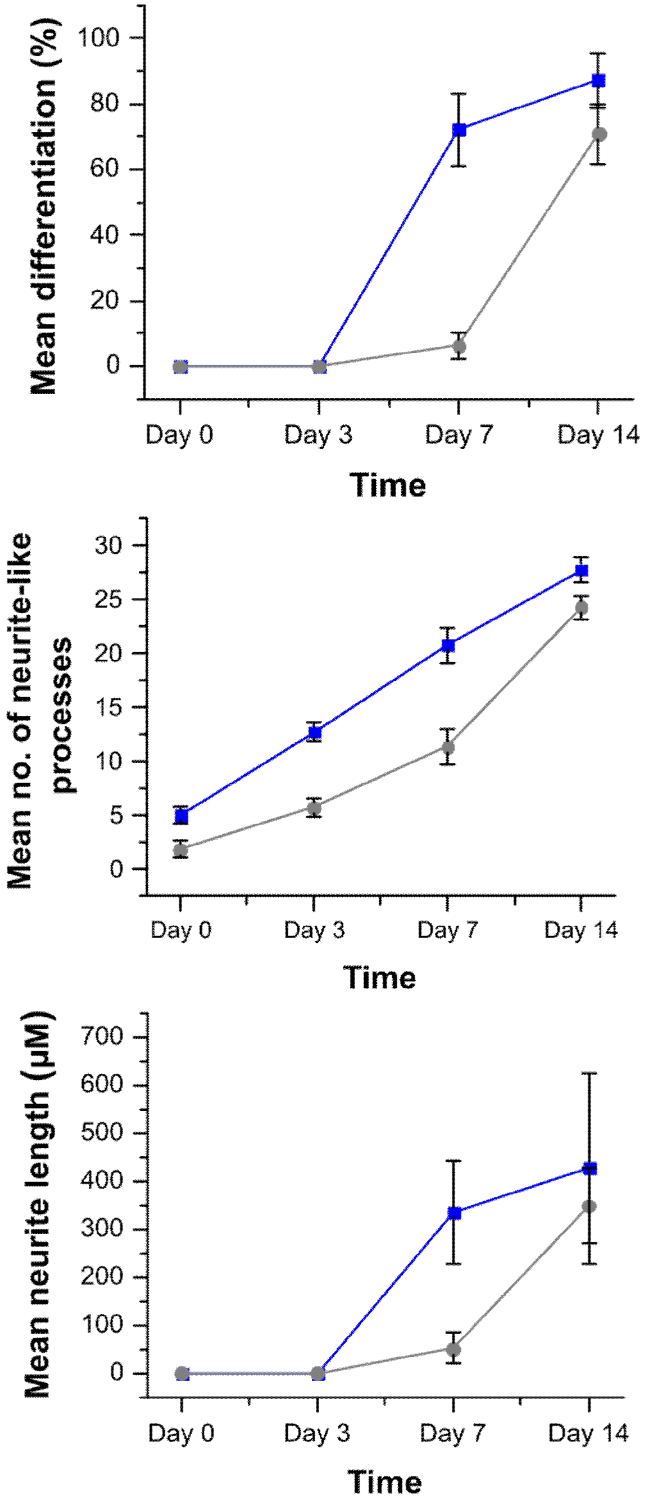

Figure S9. Differentiation of PC12 cells on hSAF hydrogels. PC12 differentiation, number of neurite-like processes and length of neural processes on *C*-terminally modified RGDS-decorated hSAF and undecorated hSAF gels (1 mM). Key: RGDS-decorated hSAF gels, blue; and undecorated hSAF gels, grey.

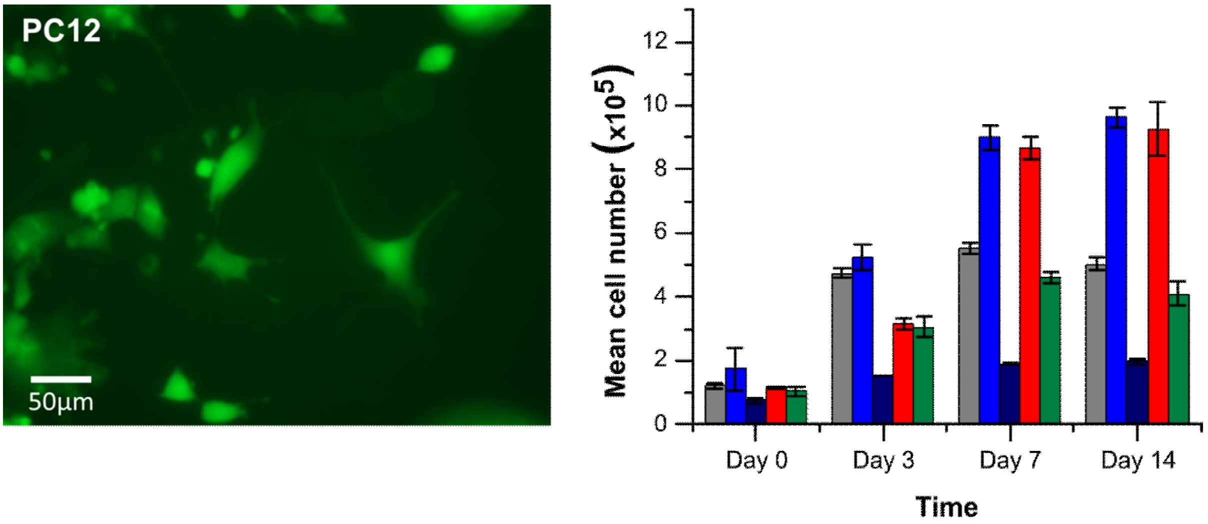

Figure S10. Cellular behavior on RGES-decorated hSAF and undecorated hSAF gels, Matrigel and TCP. Calcein-AM fluorescent image showing PC12 morphology in RGES-decorated hSAF gel. Proliferative activity of PC12 cells on these samples was assessed using MTT over 14 days. Key: undecorated hSAF gel, grey; RGDS-decorated hSAF gel, light blue; RGES-decorated hSAF gel, dark blue; Matrigel, red; and TCP, green.

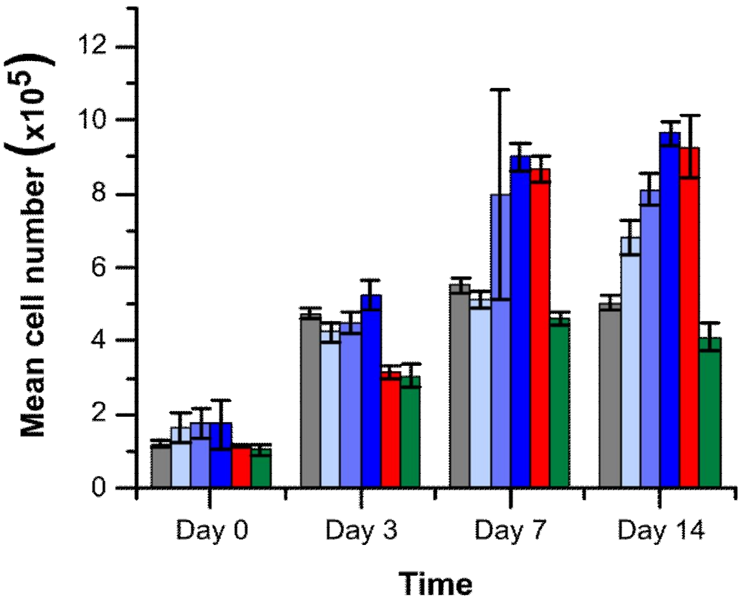

Figure S11. Proliferative activity of PC12 cells on gels comparing 1, 10 and 100% decoration with alk-RGDS. Matrigel and TCP. MTT was used to assess proliferative activity on 1-100% RGDS-decorated hSAF gels, Matrigel and TCP over 14 days. Key: undecorated hSAF gel, grey; 1% decoration, light blue; 10% decoration, purple; 100% decoration, dark blue; Matrigel, red; and TCP, green.

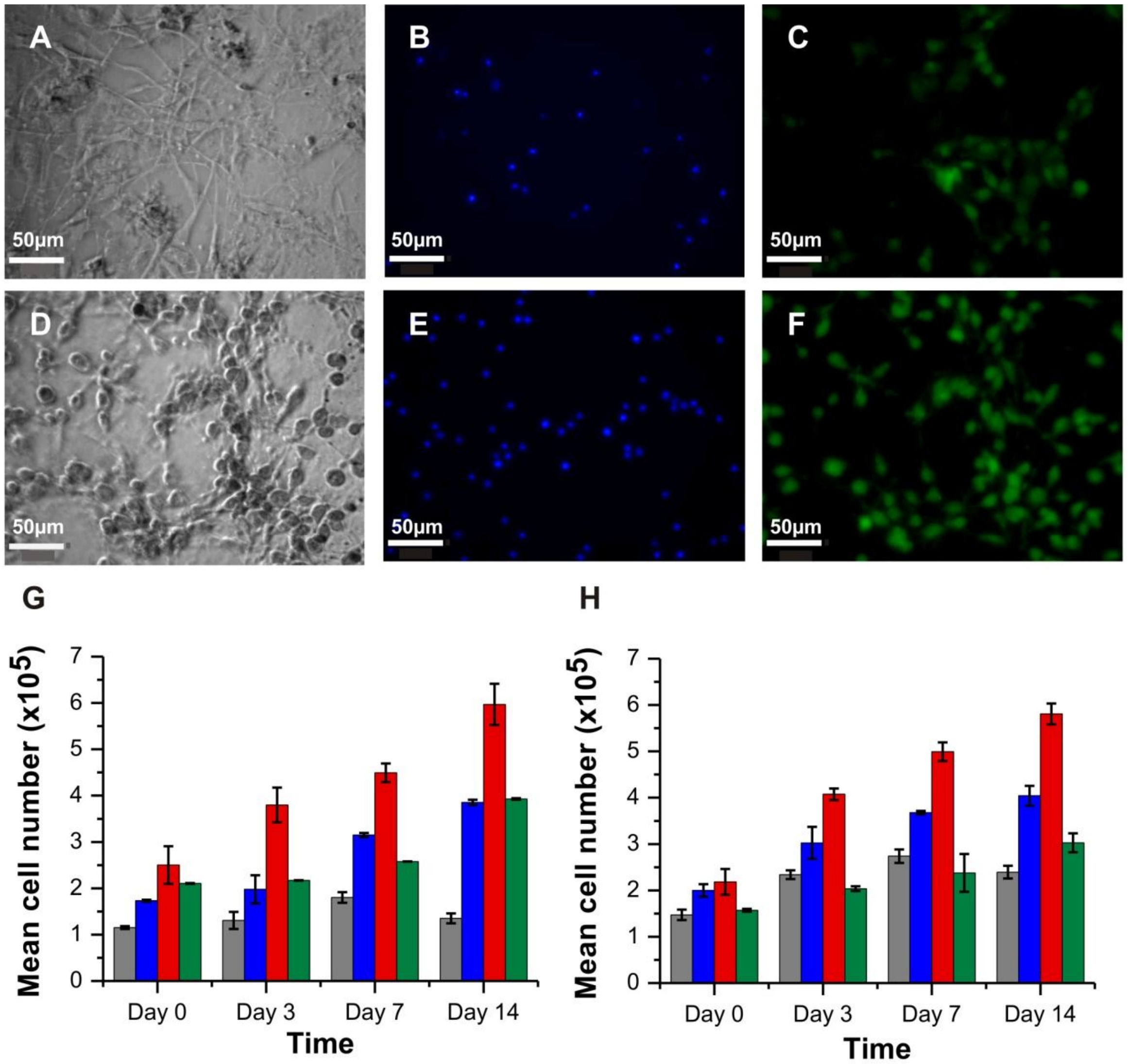

Figure S12. Attachment and proliferation of 3T3 cells on undecorated and RGDS-decorated hSAF gels, Matrigel and TCP. Light microscopy images showing fibroblasts attachment, *via* elongated cell morphology, to undecorated hSAF and RGDS-decorated hSAF gels over 14 days (A and D). Representative fluorescent images show DAPI-stained cells on undecorated hSAF and RGDS-decorated hSAF gels (B and E) with viable cells on undecorated hSAF and RGDS-decorated hSAF represented by calcein-AM staining (C and F). Proliferation of fibroblasts on the gels and TCP was quantified by measuring cell metabolic activity using MTT over 14 days (G). Hoechst dye was used to quantify the DNA of fibroblasts on the gels and TCP over 14 days (H). Key: undecorated hSAF gels, grey; RGDS-decorated hSAF gels, blue; Matrigel, red; and TCP, green.

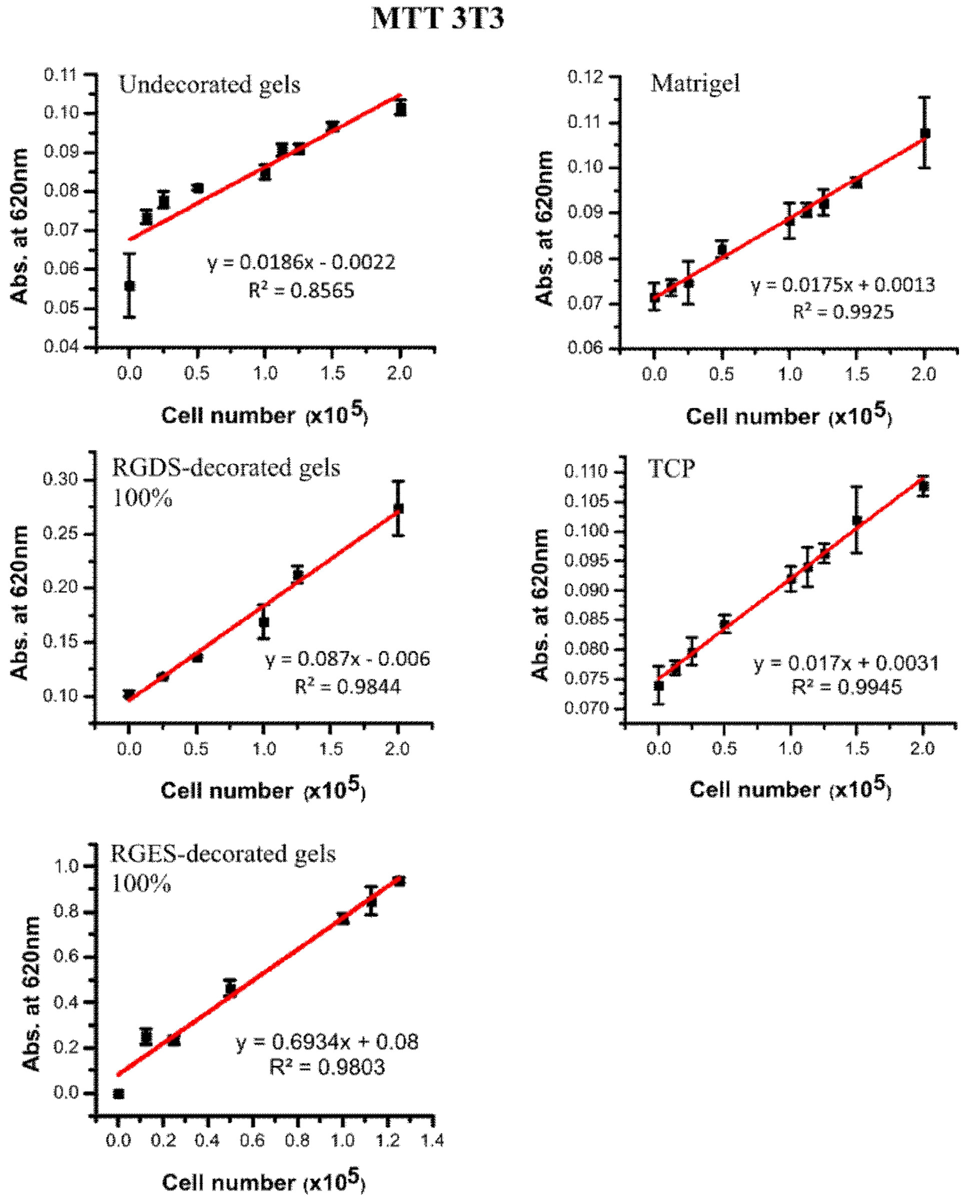

Figure S13. Metabolic activity of 3T3 cells. MTT was used to assess the metabolic activity of 3T3 cells on undecorated hSAF gels, Matrigel, 100% RGDS-decorated hSAF gels, tissue-culture plastic, and 100% RGES-decorated hSAF gels. Proliferative activity of the cells was then calculated from the calibration curves.

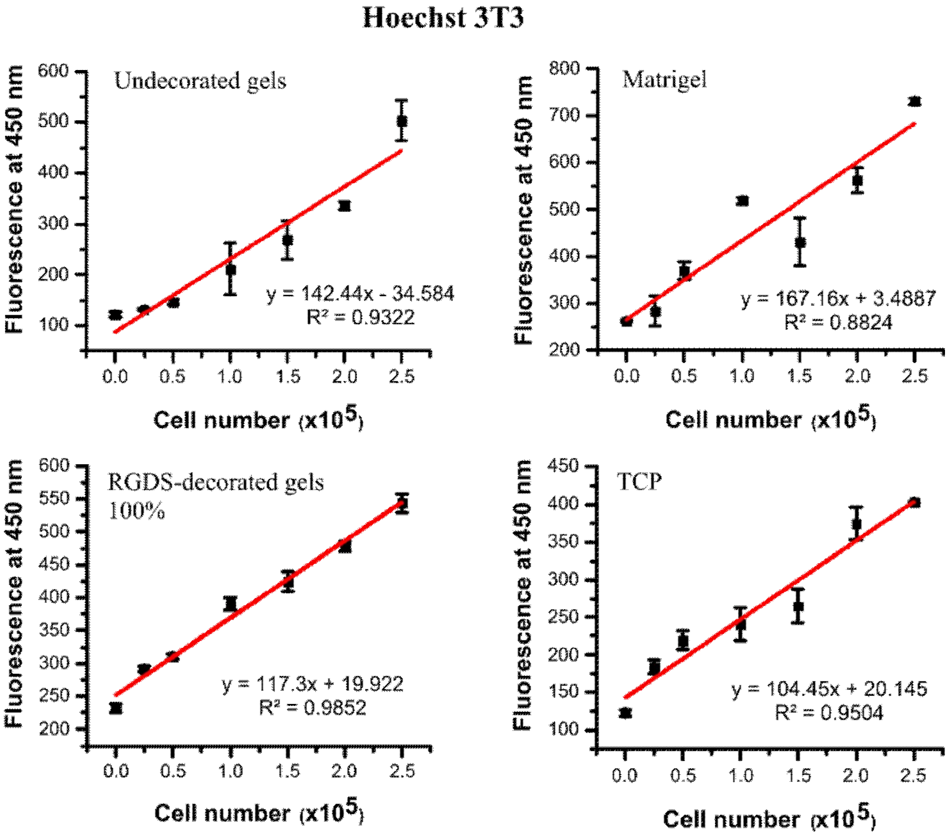

Figure S14. DNA quantification of 3T3 cells using Hoechst dye. Calibration curves of 3T3 cells stained with Hoechst dye were used to quantify the DNA content and assess approximate proliferative activity of the cells on undecorated hSAF gels, Matrigel, 100% RGDS-decorated hSAF gels, and tissue-culture plastic.
